# Supplementary material for: Chiral Recognition R- and RS- of New Antifungal: Complexation/Solubilization/Dissolution Thermodynamics and Permeability Assay
Source: Pharmaceutics. 2022 Apr 15;14(4):864. doi: 10.3390/pharmaceutics14040864 (PMC9025555; doi:10.3390/pharmaceutics14040864)
Supplement: Supplementary file 1 [file pharmaceutics-14-00864-s001.zip › pharmaceutics-1674850-supplementary.pdf]

Supporting Information

Chiral recognition R- and RS- of new antifungal: Complexation/solubilization/dissolution thermodynamics and permeability assay

Tatyana V. Volkova[a], Olga R. Simonova[a], Igor B. Levshin[b], German L. Perlovich[a*]

[a]*G.A. Krestov Institute of Solution Chemistry RAS, 153045 Ivanovo, Russia.*
[b] *Gause Institute of New Antibiotics, 119021, Moscow, Russia*
**Corresponding author:1 Akademicheskaya str., 153045 Ivanovo, Russian Federation,*
E-mail: glp@isc-ras.ru

Supporting Information - content:

Figure S1. Synthetic scheme for (a) racemic compound (RS-186) and (b) pure enantiomer (R-98). P2
Description of the synthetic procedure. P3-P4
Figure S2. ^{1}H NMR and ^{13}C NMR spectra of the studied compounds: (a) ^{1}H NMR of RS-186; (b) ^{1}H NMR of R-98; (c) ^{13}C NMR of RS-186. P5-P7
Figure S3. (a) ((5Z)-5-[(4-chlorophenyl)methylidene]-3-(2-{4-[2-(2,4-difluorophenyl)-2-hydroxy-3-(1*H*-1,2,4-triazol-1-yl)propyl]piperazin-1-yl}-2-oxoethyl)-1,3-thiazolidine-2,4-dione) (L-173); (b) fluconazole (FCZ). P8
Figure S4. PXRD patterns of raw R-98. P9
Figure S5. Temperature dependences of the molar solubility (S_2) of R-98: pH 2.0 - (a), pH 7.4 - (b), and RS-186: pH 2.0 - (c), pH 7.4 - (d) at different CD concentrations and temperatures (pressure p =100 kPa). P10-11
Figure S6. Temperature dependences of the inherent mole fraction solubility ($\ln X_2^0$) in buffer solution pH 2.0 for R-98 - black open squares and RS-186 - black open circles at pressure p =100 kPa. P12
Figure S7. Van't Hoff plots of the complex formation between R-98 (black open squares)/RS-186 (black open circles) and HP-β-CD at pH 2.0, mole fraction scale. P13
Table S1. Gibbs free energy of transfer of the compounds from pure buffer pH 2.0 to the solutions with different concentrations of CD ($\Delta G_{slbz}^{T,X}$) calculated by Eq. (10) at 4 temperatures and pressure p =100 kPa. P14

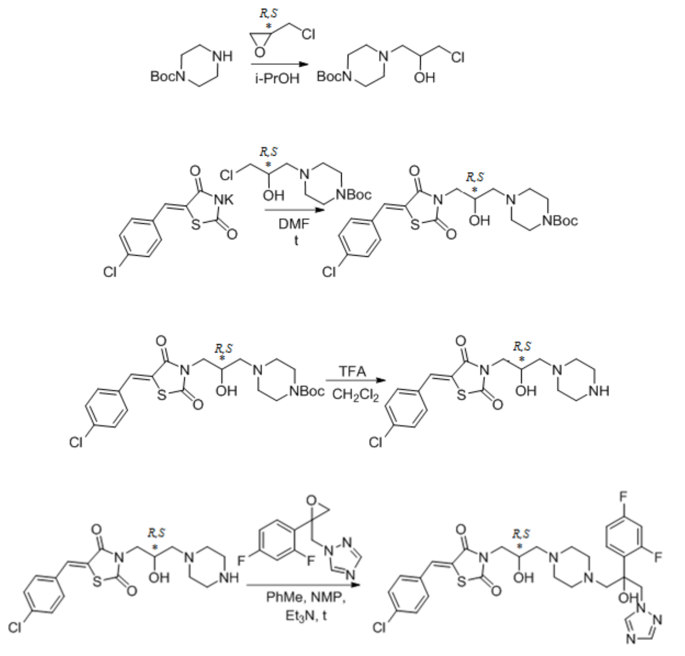

(a)

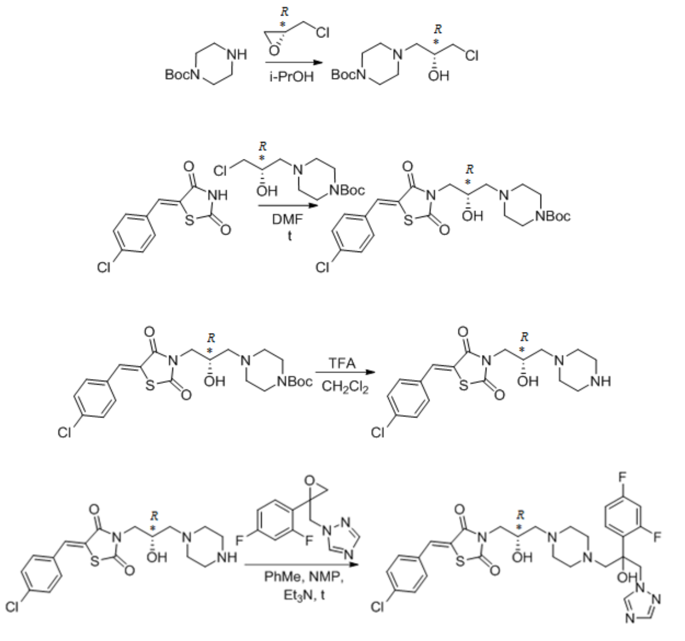

(b)

Figure S1. Synthetic scheme for (a) racemic compound (RS-186) and (b) pure enantiomer (R-98).

RS-186.

1) Synthesis of tert-butyl 4-(3-chloro-2-hydroxypropyl)piperazine-1-carboxylate.

Racemate (R,S) epichlorohydrin (2.5 g, 27 mmol) was added to a solution of tert-butylpiperazine-1-carboxylate (5.0 g, 26 mmol) in i-PrOH (50 ml). The reaction mixture was stirred overnight at room temperature, then, evaporated in vacuo to obtain (7.2 g, Yield 98%) as colorless oil. For further reactions, it was used without additional purification.

$C_{12}H_{23}ClN_2O_3$. m/z: 278.78. LC-MS 279 $(M+1)^+$.

^{1}H NMR (500 MHz, DMSO-d6): δ ppm 1.42 (s, 9 H), 2.27-2.42 (m,6 H), 3.22-3.32 (m, 4 H), 3.52-3.56 (m, 1H), 3.79-3/89 (m, 1H), 5.04 (d, J= 4.9 Hz, 1H).

2) Synthesis of tert-butyl (Z)-4-(3-(5-(4-chlorobenzylidene)-2,4-dioxothiazolidin-3-yl)-2-hydroxypropyl)piperazine-1-carboxylate.

Potassium salt of (Z)-5-(4-chlorobenzylidene)thiazolidine-2,4-dione was suspended to a solution of tert-butyl 4-(3-chloro-2-hydroxypropyl)piperazine-1-carboxylate (4.0 g, 14 mmol) in dry DMF (50 ml). The reaction mixture was vigorously stirred at 90 °C for 12 hours, then cooled to room temperature and evaporated in vacuo. The solid residue was mixed with water (50 ml) and EtOAc (100 ml). The organic extract was separated, washed with water (20 ml), the solvent was partially evaporated (up to 1/2 volume) and cooled to +5 °C. The precipitate was filtered, washed with i-PrOH (5 ml), and dried at room temperature. (R,S) tert-butyl(Z)-4-(3-(5-(4-chlorobenzylidene)-2,4-dioxothiazolidin-3-yl)-2-hydroxypropyl)piperazine-1-carboxylate (5.12 g , Yield 74%) as colorless amorphous powder was obtained.

$C_{22}H_{28}ClN_3O_5S$. Exact mass: 481.1438. LC-MS m/z: 481.8 $(M+1)^+$.

^{1}H NMR (500 MHz, DMSO-d6): δ ppm 1.32- 1.43 (m, 9 H) 2.20-2.44 (m, 6 H) 2.24-2.42 (m, 1 H) 3.30 (br s, 4 H+water) 3.63-3.76 (m, 2 H) 3.89-4.04 (m, 1 H) 5.06 (br d, J=4.77 Hz, 1 H),7.56-7.69 (m, 4 H) ,7.92 (s, 1 H).

3) Synthesis of (Z)-5-(4-chlorobenzylidene)-3-(2-hydroxy-3-(piperazin-1-yl)propyl)thiazolidine-2,4-dione.

TFA (10 ml, 0.1 mol) was added to a solution of tert-butyl (S,Z)-4-(3-(5-(4-chlorobenzylidene)-2,4-dioxothiazolidin-3-yl)-2-hydroxypropyl)piperazine-1-carboxylate (4.5 g. 9.3 mmol) in CH_2Cl_2 (50 ml). The reaction mixture was stirred at room temperature for 5 hours, then, evaporated in vacuo. Water (50 ml) and EtOAc (100 ml) were added to the dry residue and then Na_2CO_3 (2.1 g, 20 mmol) was added carefully upon stirring. When the neutralization process was finished, the organic layer was separated, the aqueous layer was re-extracted with EtOAc (50 мл). The extracts were combined and washed with brine, dried over anhydrous Na_2SO_4, evaporated in vacuo. (R,S) (Z)-5-(4-chlorobenzylidene)-3-(2-hydroxy-3-(piperazin-1-yl)propyl) thiazolidine-2,4-dione (3.5 g, Yield 98%) in the form of a colorless oil was obtained.

$C_{17}H_{20}ClN_3O_3S$. Exact mass: 381,0914. LC MS m/z: $(M+1)^+$ =381.8$^+$.

^{1}H NMR (500 MHz, DMSO-d6) δ ppm 2.07-2.42 (m, 4 H), 2.52-2.80 (m, 2 H) 3.41 (br dd, J=1.79, 1.05 Hz, 6H+water), 3.56-3.75 (m, 3 H), 3.85-4.08 (m, 1 H), 7.48-7.70 (m, 4 H), 7.90 (s, 1 H).

4) Synthesis of (R,S) (Z)-5-(4-chlorobenzylidene)-3-(3-(4-(2-(2,4-difluorophenyl)-2-hydroxy-3- (1H-1,2,4 -triazol-1-yl)propyl)piperazin-1-yl)-2-hydroxypropyl)thiazolidine-2,4-dione.

1-((2-(2,4-difluorophenyl)oxiran-2-yl)methyl)-1H-1,2,4-triazole (1.9 g, 8 mmol) and Et_3N (5 ml, 38 mmol) were added to a solution of (Z)-5-(4-chlorobenzylidene)-3-(2-hydroxy-3-(piperazin-1-yl)propyl)thiazolidine-2,4-dione (3.0 g, 7.8 mmol) in a mixture PhMe: NMP - 20:1 (100 ml). The reaction mixture was stirred at a temperature of 90 °C for 12 hours, then

cooled to room temperature and evaporated in vacuum. The residue was purified by flesh chromatography using EtOAc:MeOH:Et_3N (25:1:0.1) as eluent.

(R,S) (Z)-5-(4-chlorobenzylidene)-3-(3-(4-(2-(2,4-difluorophenyl)-2-hydroxy- 3-(1H-1,2,4-triazol-1-yl)propyl)piperazin-1-yl)-2-hydroxypropyl) thiazolidine-2,4-dione (3.2 g, Yield 67%) as amorphous powder was obtained.

$C_{28}H_{29}ClF_2N_6O_4S$. Exact mass: 618.1628. LC MS m/z $(M+1)^+ = 619.1^+$, HPLC-MS, column Phenomenex C18, 150 *3.3 μm, column temperature 30 °C (mobile phase: acetonitrile), m/z 619.1810, retention time: 10.4-10.5 min.

R-98.

1) Synthesis of tert-butyl 4-(3-chloro-2-hydroxypropyl)piperazine-1-carboxylate.

Prepared from tert-butylpiperazine-1-carboxylate and (R)-epichlorohydrin according to the method described above for RS-186. Obtained: (4.9 g, Yield 97%) as colorless oil. Then it was used without additional purification.

$C_{12}H_{23}ClN_2O_3$. m/z: 278.776. LC-MS: 278.9 $(M+1)^+$.

2) Synthesis of tert-butyl (Z)-4-(3-(5-(4-chlorobenzylidene)- 2,4-dioxothiazolidin-3-yl)-2-hydroxypropyl)piperazine-1-carboxylate. The compound was prepared from tert-butyl (R)-4-(3-chloro-2-hydroxypropyl)piperazine-1-carboxylate as described above for RS-186. (R) (Z) tert-butyl 4-(3-(5-(4-chlorobenzylidene)-2,4-dioxothiazolidin-3-yl)-2-hydroxypropyl)piperazine-1-carboxylate (5.26 g, Yield 78 %) as colorless amorphous powder was obtained.

$C_{22}H_{28}ClN_3O_5S$. Exact mass: 481.1438. LC-MS m/z: 481.8 (M+1)+.

^1H NMR (500 MHz, DMSO-d6) δ ppm 1.32-1.43 (m, 9 H), 2.20-2.44 (m, 6 H), 2.24-2.42 (m, 1H), 3.30 (br s, 4 H+water), 3.63-3.76 (m, 2 H), 3.89-4.04 (m, 1 H), 5.06 (br d, J=4.77 Hz, 1 H), 7.56-7.69 (m, 4 H) ,7.92 (s, 1 H).

3) Synthesis of (Z)-5-(4-chlorobenzylidene)-3-(2-hydroxy-3-(piperazin-1-yl)propyl)thiazolidine-2,4-dione. Prepared from tert-butyl (R)-4-(3-chloro-2-hydroxypropyl) piperazine-1-carboxylate as described above for RS-186. (R) (Z)-5-(4-chlorobenzylidene)-3-(2-hydroxy-3-(piperazin-1-yl)propyl)thiazolidine-2,4-dione (3.5 g, Yield 98%) was obtained as colorless oil.

$C_{17}H_{20}ClN_3O_3S$. LC MS m/z: $(M+1)^+=381.8^+$.

4) Synthesis of (R) (Z)-5-(4-chlorobenzylidene)-3-(3-(4-(2-(2,4-difluorophenyl)-2-hydroxy-3- (1H-1,2,4 -triazol-1-yl)propyl)piperazin-1-yl)-2-hydroxypropyl) thiazolidine-2,4-dione.

Prepared from (R) (Z)-5-(4-chlorobenzylidene)-3-(2-hydroxy-3-(piperazin-1-yl)propyl)thiozolidine-2,4-dione according to the method described above for RS-186. R-isomer (3.5 g, Yield 73%) was obtained as colorless amorphous powder.

$C_{28}H_{29}ClF_2N_6O_4S$. Exact Mass: 381.0914. LC MS m/z: $(M+1)^+=381.8^+$

The degree of purity estimated using HPLC (Shimadzu LC-20 AD, System - FOC Colon- Kromasil -100-5mkm. C18, 4.6x250 mm., N 62511. Elution: A - H_3PO_4 0.01 pH 2.6; B - MeCN, fl -1.0 ml/min, loop 20 mkl, and Phenomenex column C18, 150*, 3.3 μm, column temperature 30°C (mobile phase: acetonitrile), m/z 619,1810; retention time: 10.4- 10.5 min.) was 96% and 97% for the RS-186 and R-98 components. The purity was additionally approved by ^1H NMR, ^{13}C NMR spectra.

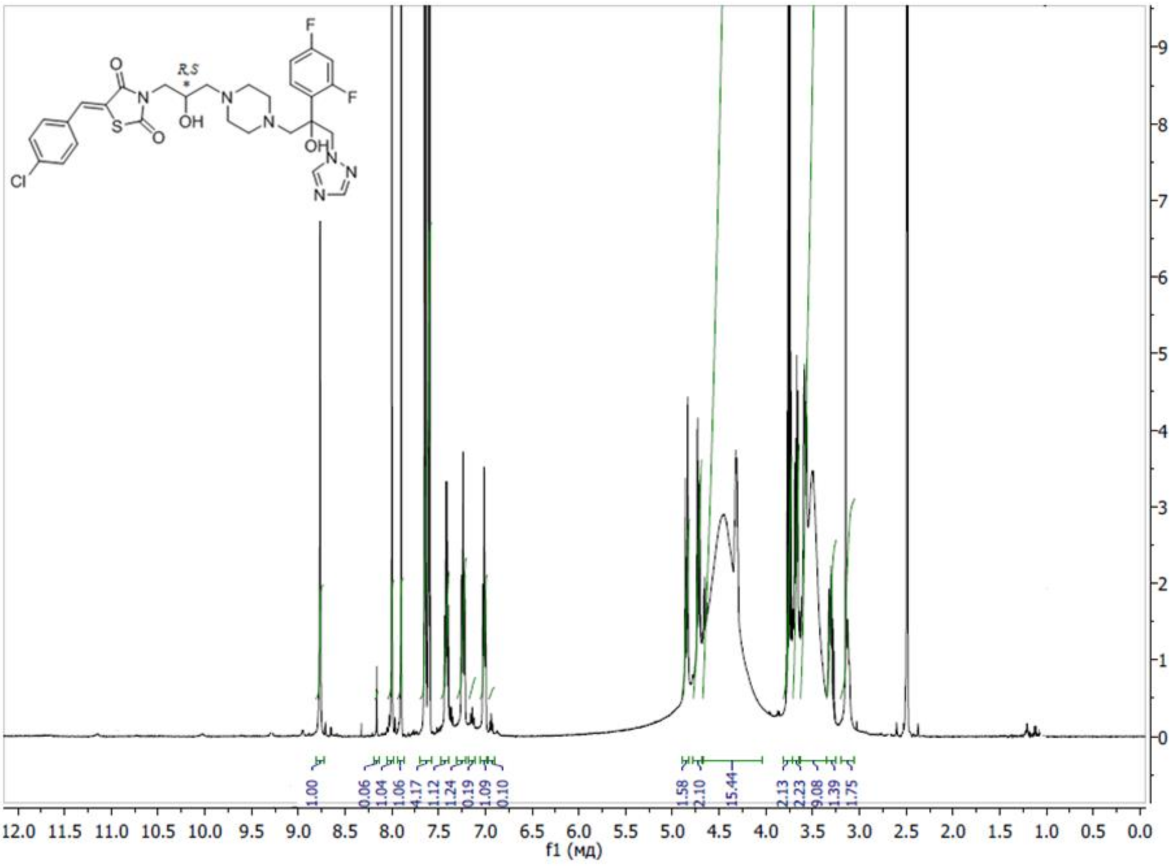

(a)

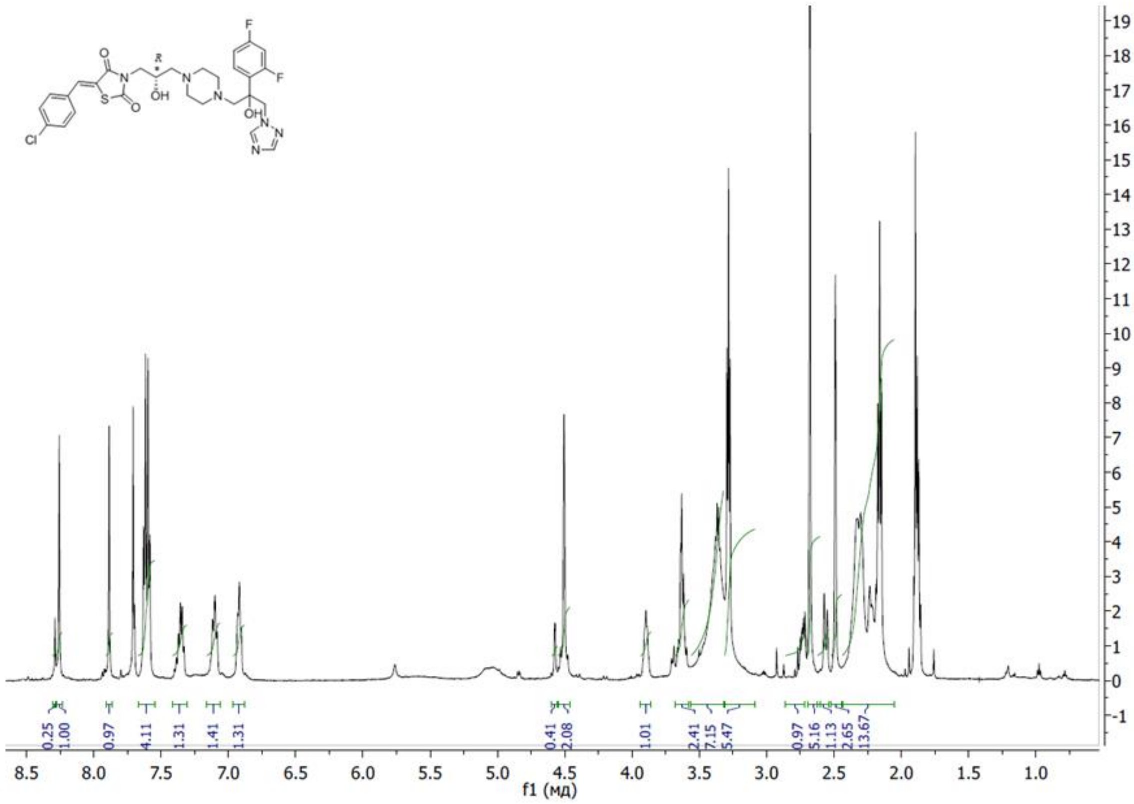

Cl
S
O
N
R
OH
F
OH
N
N
N
0.25
1.00
0.97
4.11
1.31
1.41
1.31
0.41
2.08
1.01
2.41
7.15
5.47
0.97
5.16
1.13
2.65
13.67
f1 (мд)

(b)

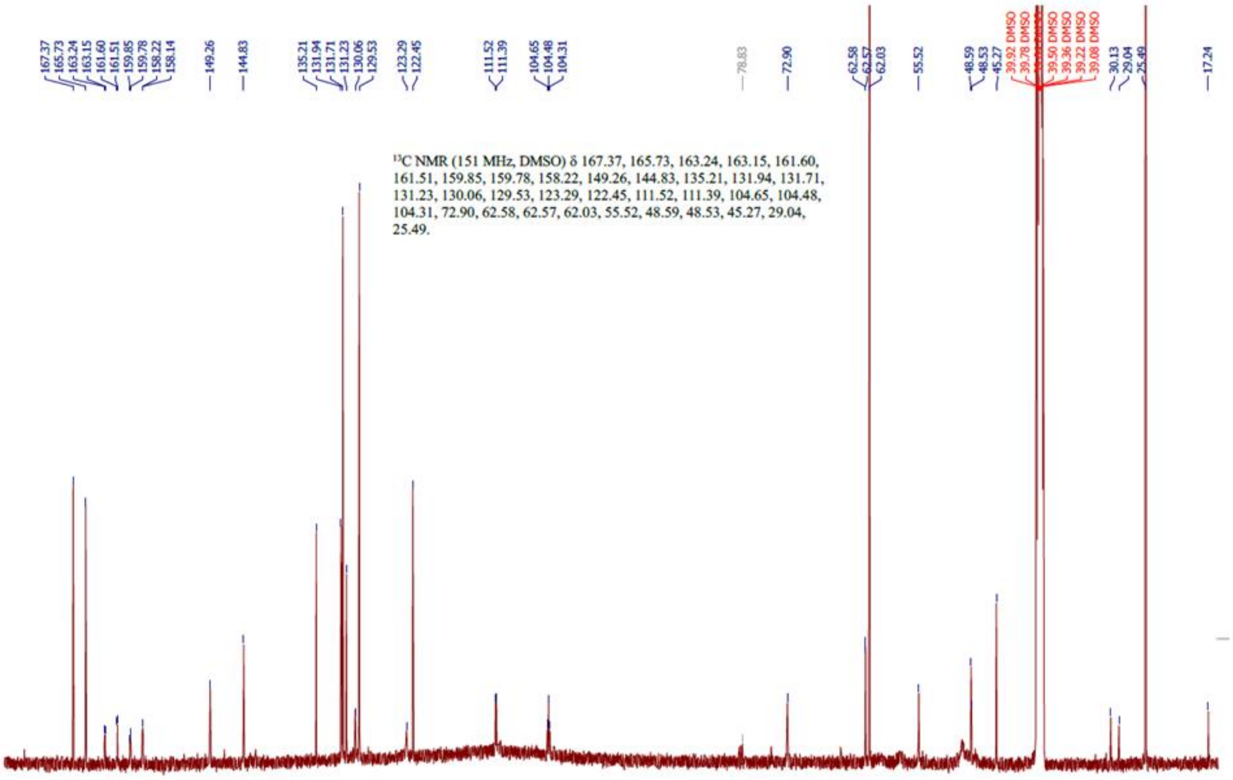

(c)

Figure S2. ^{1}H NMR and ^{13}C NMR spectra of the studied compounds: (a) ^{1}H NMR of RS-186; (b) ^{1}H NMR of R-98; (c) ^{13}C NMR of RS-186.

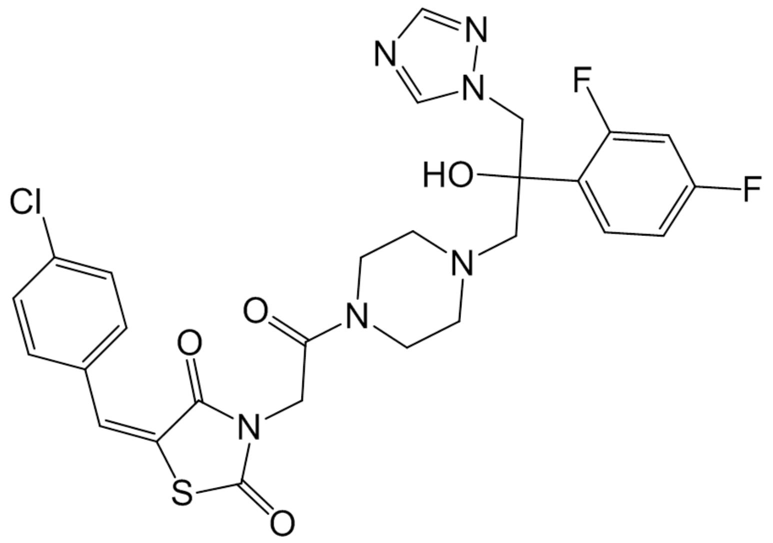

(a)

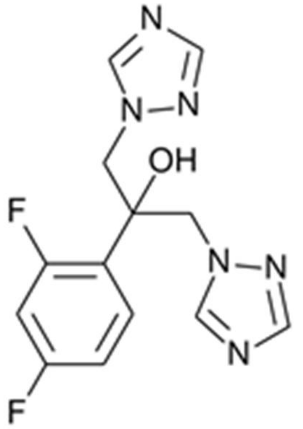

(b)

Figure S3. (a) ((5Z)-5-[(4-chlorophenyl)methylidene]-3-(2-{4-[2-(2,4-difluorophenyl)-2-hydroxy-3-(1*H*-1,2,4-triazol-1-yl)propyl]piperazin-1-yl}-2-oxoethyl)-1,3-thiazolidine-2,4-dione) (L-173); (b) fluconazole (FCZ).

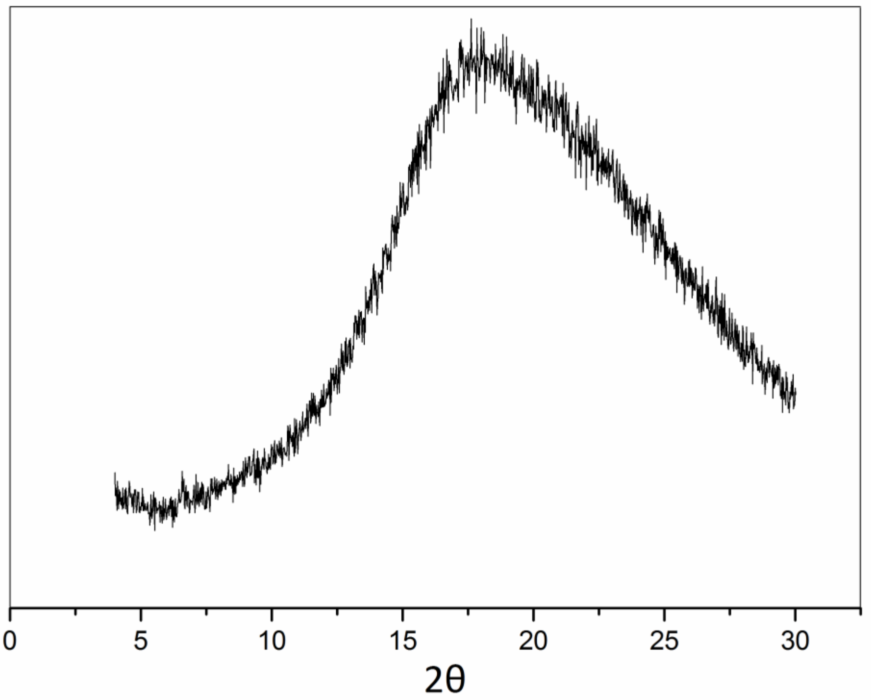

Figure S4. PXRD patterns of raw R-98.

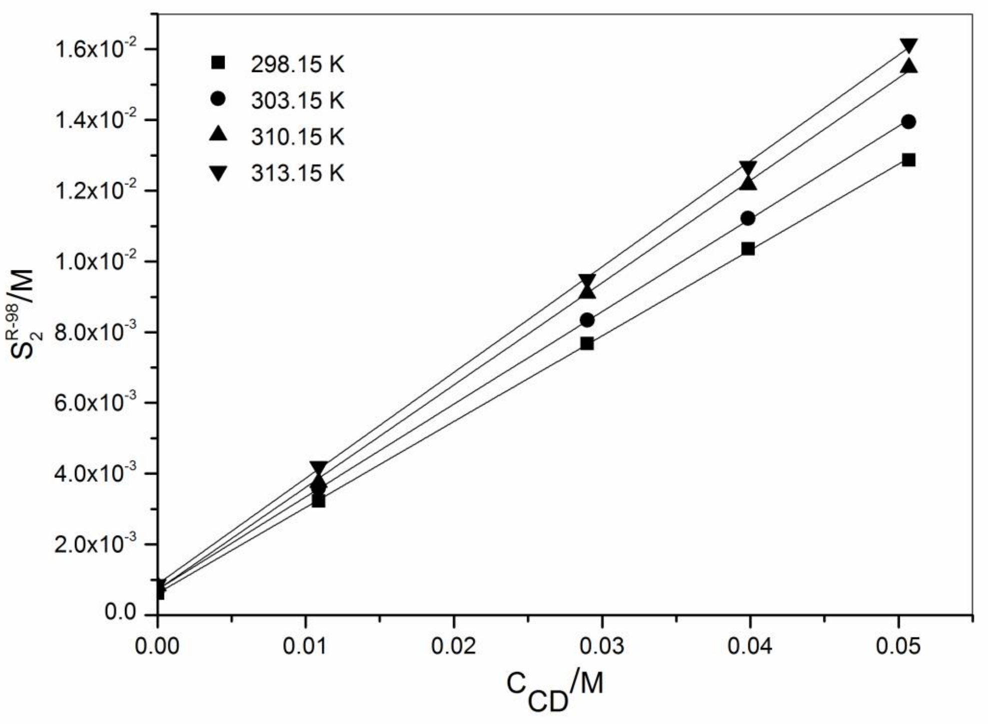

298.15 K
303.15 K
310.15 K
313.15 K
S_2^{R-98}/M
C_{CD}/M

(a)

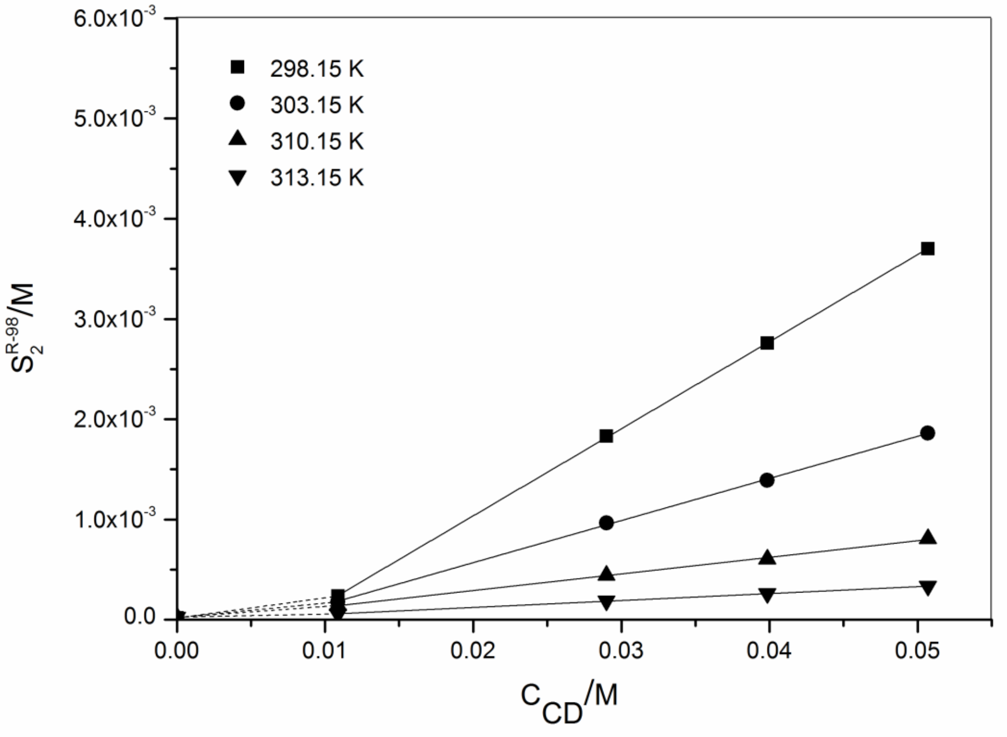

298.15 K
303.15 K
310.15 K
313.15 K
S_2^{R-98}/M
C_{CD}/M

(b)

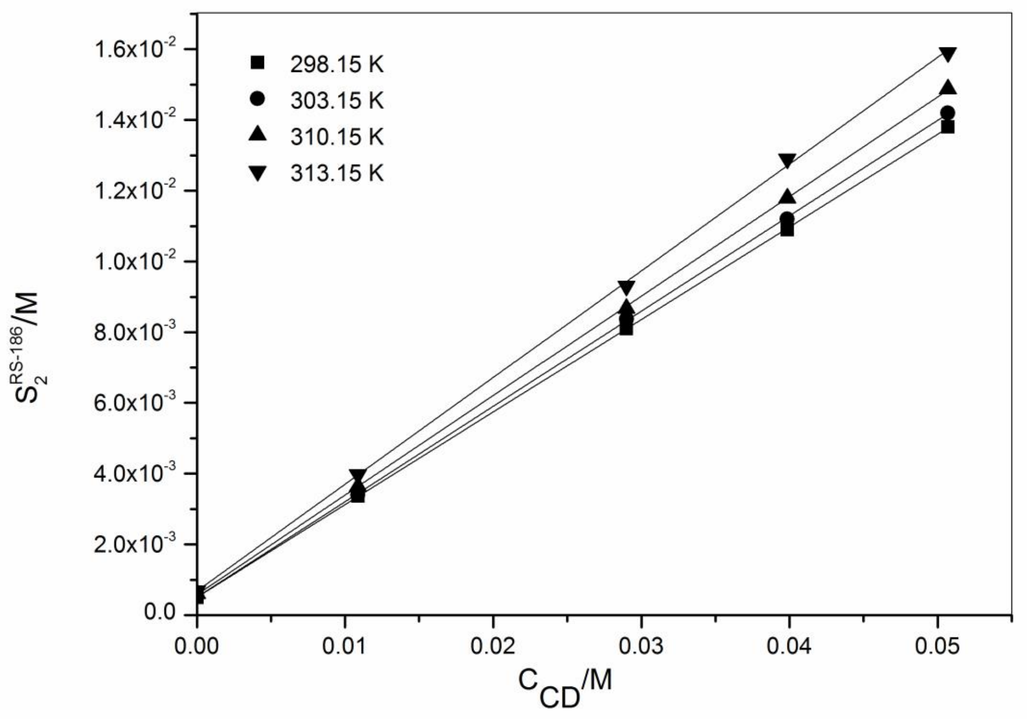

(c)

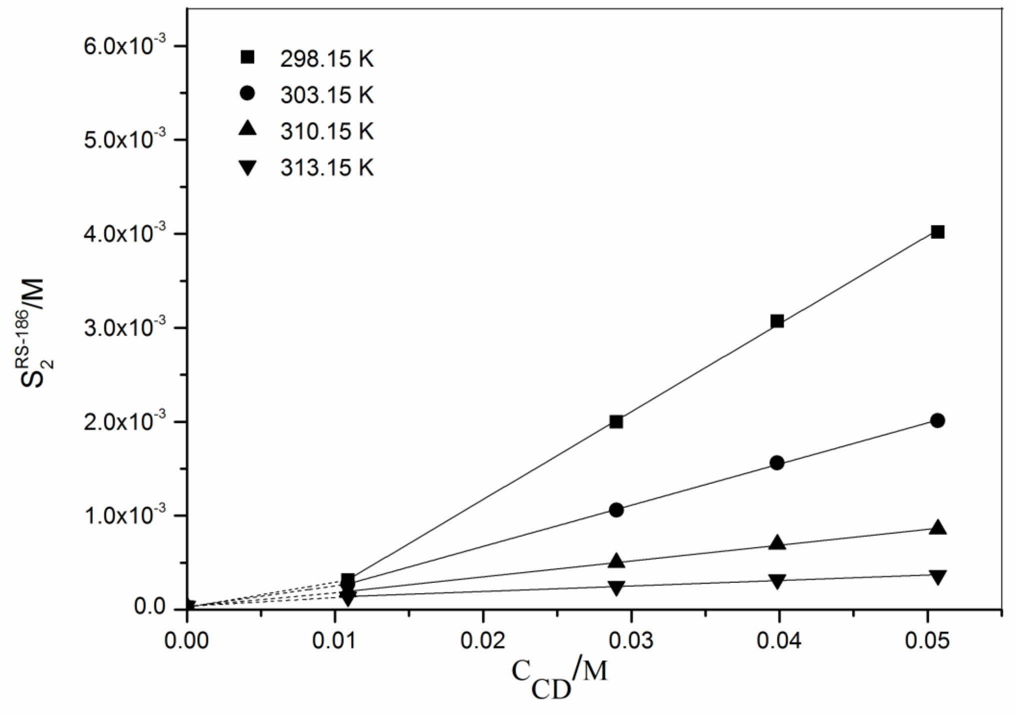

(d)

Figure S5. Temperature dependences of the molar solubility (S_2) of R-98: pH 2.0 - (a), pH 7.4 - (b), and RS-186: pH 2.0 - (c), pH 7.4 - (d) at different CD concentrations and temperatures (pressure p =100 kPa).

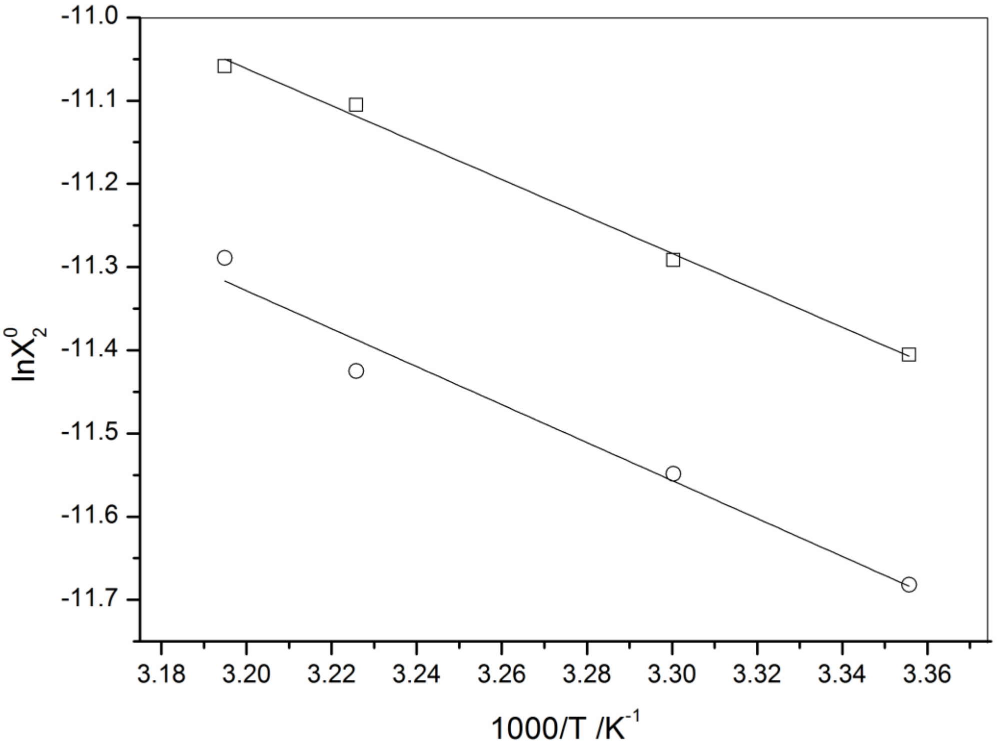

Figure S6. Temperature dependences of the inherent mole fraction solubility ($\ln X_2^0$) in buffer solution pH 2.0 for R-98 - black open squares and RS-186 - black open circles at pressure *p* =100 kPa.

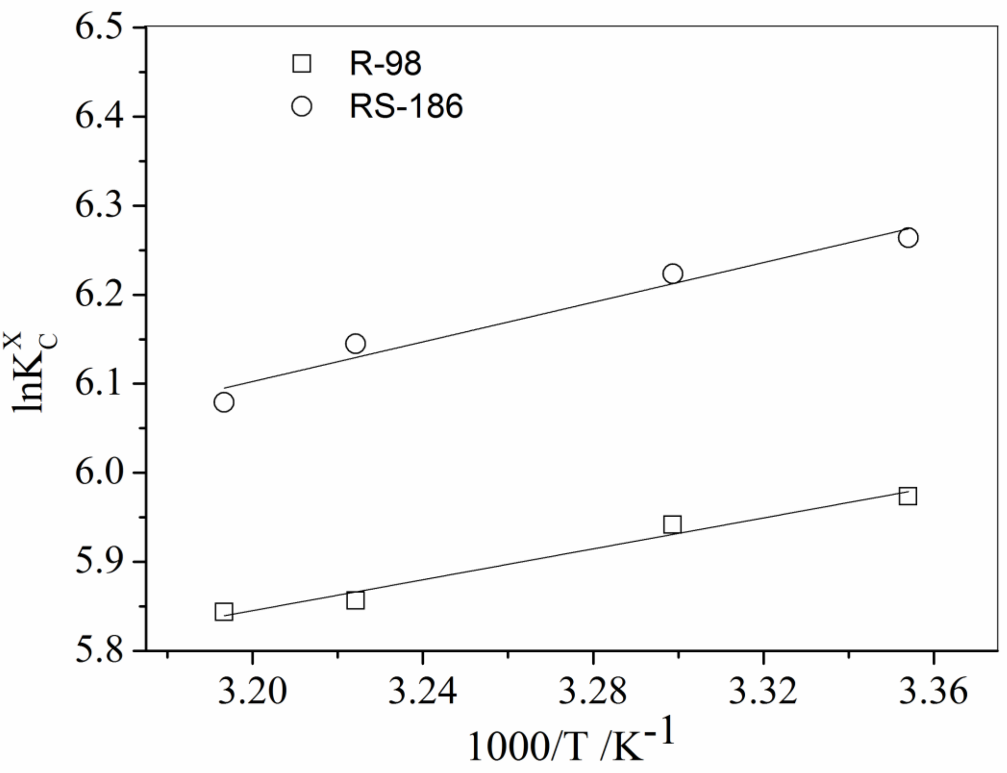

Figure S7. Van't Hoff plots of the complex formation between R-98 (black open squares)/RS-186 (black open circles) and HP-β-CD at pH 2.0, mole fraction scale.

Table S1. Gibbs free energy of transfer of the compounds from pure buffer pH 2.0 to the solutions with different concentrations of CD ($\Delta G_{slbz}^{T,X}$) calculated by Eq. (10) at 4 temperatures and pressure *p* =100 kPa.

T/K	298.15 K	303.15 K	310.15 K	313.15 K
C_{CD}		R-98		
0.01	-4.09	-4.43	-4.64	-5.01
0.03	-6.24	-6.55	-6.93	-7.15
0.04	-6.98	-7.30	-7.68	-7.91
0.05	-7.52	-7.85	-8.30	-8.54
C_{CD}		RS-186		
0.01	-4.73	-4.83	-5.12	-5.43
0.03	-6.90	-7.10	-7.36	-7.66
0.04	-7.64	-7.84	-8.15	-8.52
0.05	-8.22	-8.43	-8.75	-9.06
